# The Ongoing Epidemic of West Nile Virus in Greece: The Contribution of Biological Vectors and Reservoirs and the Importance of Climate and Socioeconomic Factors Revisited

**DOI:** 10.3390/tropicalmed8090453

**Published:** 2023-09-19

**Authors:** Dimitrios Kouroupis, Konstantina Charisi, Athina Pyrpasopoulou

**Affiliations:** 1 2nd Propedeutic Department of Internal Medicine, Hippokration Hospital, Konstantinoupoleos 49, 54642 Thessaloniki, Greece; dimcour841@gmail.com; 2 Infectious Diseases Unit, Hippokration Hospital, Konstantinoupoleos 49, 54642 Thessaloniki, Greece; konstantina26@hotmail.com

**Keywords:** WNV, emerging infectious disease, Greece, endemic

## Abstract

Emerging infectious diseases have inflicted a significant health and socioeconomic burden upon the global population and governments worldwide. West Nile virus, a zoonotic, mosquito-borne flavivirus, was originally isolated in 1937 from a febrile patient in the West Nile Province of Uganda. It remained confined mainly to Africa, the Middle East, and parts of Europe and Australia until 1999, circulating in an enzootic mosquito-bird transmission cycle. Since the beginning of the 21st century, a new, neurotropic, more virulent strain was isolated from human outbreaks initially occurring in North America and later expanding to South and South-eastern Europe. Since 2010, when the first epidemic was recorded in Greece, annual incidence has fluctuated significantly. A variety of environmental, biological and socioeconomic factors have been globally addressed as potential regulators of the anticipated intensity of the annual incidence rate; circulation within the zoonotic reservoirs, recruitment and adaptation of new potent arthropod vectors, average winter and summer temperatures, precipitation during the early summer months, and socioeconomic factors, such as the emergence and progression of urbanization and the development of densely populated areas in association with insufficient health policy measures. This paper presents a review of the biological and socioenvironmental factors influencing the dynamics of the epidemics of West Nile virus (WNV) cases in Greece, one of the highest-ranked European countries in terms of annual incidence. To date, WNV remains an unpredictable opponent as is also the case with other emerging infectious diseases, forcing the National Health systems to develop response strategies, control the number of infections, and shorten the duration of the epidemics, thus minimizing the impact on human and material resources.

## 1. West Nile Virus: Pathogenicity and Virulence

West Nile virus (WNV), is a positive-stranded ribonucleic acid (RNA) virus. It belongs to the family of Flaviviridae, in the Japanese encephalitis antigenic complex [1]. Other members of the family include Zika virus, Dengue virus, and Yellow Fever virus. West Nile virions are ~50 nm in diameter with icosahedral symmetry and consist of a spherical nucleocapsid surrounded by an envelope [2].

WNV was first isolated in 1937 from a woman in the West Nile district in Uganda [3]. In the 77 years since its discovery, the virus has expanded to a vast region of the globe, in parts of North and South America, Africa, Europe, Asia, and Oceania, and is now considered the most important causative agent of viral encephalitis worldwide [4].

Eight phylogenetic lineages have been described, but only lineages 1 and 2 are associated with disease in humans. Lineage 1 and its multiple profiles are the source of the epidemic transmission in Africa and throughout the world. Lineage 2 was considered an African zoonosis and has historically been considered to be less pathogenic in humans than lineage 1. However, in 2004, lineage 2, previously only isolated from horses in sub-Saharan Africa and Madagascar, appeared in Hungary, where the first recorded outbreak affected 18 horses. Prior to the emergence of a lineage 2 strain in Hungary in 2004, sporadic cases and occasional outbreaks in animals and humans in Europe were linked to lineage 1 strains. Since 2008, the lineage 2 strain has spread over central Europe and the eastern Mediterranean region and has become more virulent in vertebrates [5]. This strain, although not previously considered to be of public health importance, appears to have evolved into a more virulent phenotype and has since caused significant outbreaks in humans and animals in several countries, including Greece, Hungary, and Serbia [6]. A discrete lineage 2 virus emerged in southern Russia in 2007, which spread to Romania and Italy after 2010.

The significant majority of WNV infections in humans are asymptomatic. About 20–25% of WNV infections may cause a mild disease, associated with a wide variety of symptoms [7]. Neuroinvasive disease (meningitis, encephalitis, acute flaccid paralysis), although very rare, occurring in <1% of the infected patients, is characterised by significant mortality (~10%) and long-term morbidity [8] and occurs more readily in aged and immunocompromised patients.

Infection is initiated after the vector salivary components are introduced into keratinocytes and skin dendritic cells and/or macrophages. The inflammatory response that follows leads to the recruitment of neutrophils, which secrete attractants to summon monocytes. The monocytes are differentiated into dendritic cells and macrophages and become attacked by the virus, while in the meantime, the resident dermal dendritic cells migrate to draining lymph nodes to induce adaptive immunity [9]. In the cases of neuroinvasive disease, the virus is considered to cross the blood-brain barrier either directly, or indirectly, through infected endothelial cells, macrophages, or the axons of olfactory or peripheral neurons [10]. WNF is characterised by a sudden onset of symptoms that may include headache, malaise, fever, myalgia, vomiting, papular rash, fatigue, and eye pain [11]. Symptom severity ranges from a mild self-limiting illness from which patients recover within one week to protracted fatigue and malaise that can last for months. The cornerstone of diagnosis remains positive serology for acute infection that usually converts after the first week of disease initiation [12]. Viremia, like in the case of other flaviviruses, is of low levels and of short duration. Treatment is symptomatic; while effective vaccines have been licensed for the prophylaxis of equids, no FDA-approved vaccine exists for human use yet [13].

## 2. Transmission Vectors, Life Cycle and Animal Hosts of WNV

**Scheme 1:**
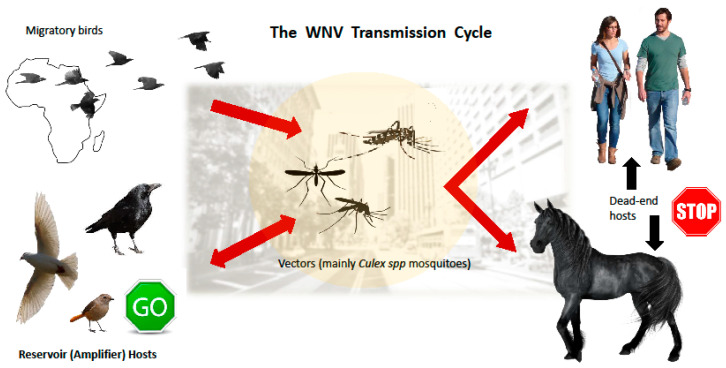
The WNV Transmission Cycle: The virus circulates between vectors (mainly *Culex* spp. mosquitoes and the reservoir hosts; it accidentaly infects dead-end hosts (humans, equids).

WNV is maintained in nature in an enzootic transmission cycle between avian hosts and ornithophilic mosquito vectors. Besides the main hosts/reservoirs which will develop WNV disease, outbreaks may develop in horses and humans, with systemic symptomatology [14]. They become infected accidentally and represent dead-end hosts (Scheme 1). The virus is transmitted almost exclusively by mosquitoes, mostly species of *Culex*, along with the Japanese encephalitis and Murray Valley encephalitis viruses [15]. Although of indeterminate significance, WNV has been detected in ticks (Hyalomma marginatum marginatum nymph) [16]. In Europe, the ornithophilic mosquito species *Culex pipiens* (biotype) *pipiens* and *Culex pipiens* (biotype) *molestus* are the main vectors of WNV south of the Alps, with *Culex torrentium* being the dominant species in northern Europe [17]. 

Besides Culicidae, experimental data additionally confirmed the capacity of *Aedes* and other mosquitoes to transmit WNV [18]; in fact, efficient transmission has been experimentally demonstrated with most of the evaluated mosquito species. Being an efficient vector requires regular feeding on competent hosts. *Aedes albopictus*, an invasive tiger mosquito with behavioural characteristics which render it a public health threat in terms of potential contribution to the manifestation of emerging infectious disease outbreaks, is proven to be a competent vector in laboratory settings. In praxis, it is considered to be of little importance for transmission as these species mainly bite mammals and are thus unlikely to acquire the infection from birds [19,20]. In contrast, the *Culex pipiens* (biotype) *pipiens* prefers feeding on birds and plays an important role in the enzootic transmission cycle of WNV. 

## 3. WNV Disease: An Emerging or Re-Emerging Infectious Disease?

According to serological surveys, WNV has circulated in Europe since the 1950s [21], and the first recognized human outbreak was recorded in 1962–1963 in Camargue, Southern France [22]. However, up until 1996, the cases that were reported were sporadic. In the past two decades, outbreaks have been recorded practically annually, both in animals as well as humans (ECDC, Historical data by year—West Nile virus seasonal surveillance). Several epidemiological studies have tried to address the causes for its evolution to an emerging infectious disease and have focused both on inherent viral factors as well as extrinsic (environmental, vector-associated and/or human) ones.

The sustainability of WNF outbreaks in South-eastern Europe since 2010 presumes the overwintering of the virus in the region. In support of this theory, the same WNV strain that was identified during the 2010 outbreak in Greece was isolated from sentinel chickens in 2011 from neighbouring regions, indicating the ability of the virus to overwinter in this region [23]. Additionally, viral seropositivity of non-migratory bird species would suggest that these may provide a reservoir for infection of mosquitoes and further transmission of the virus or other avian species which thus do not need to migrate to Africa to become infected [24].

The major factor currently favouring viral overwintering in contrast to previous decades appears to be the rise of the median winter temperatures to levels permissive for vector survival [25]. The theory that the introduction of new vectors, such as the *Aedes* tiger mosquitoes which are adapted to humanized urbanized environments, would have contributed significantly is not supported by the current evidence.

## 4. Climate Factor Deviations and Mosquito-Borne Disease Expansion 

Climate change is considered to be a major contributing factor to the global expansion of mosquito-borne diseases, previously restricted to tropical and subtropical areas [26]. Outbreaks of Chikungunya and West Nile virus disease, and autochthonous cases of Dengue fever and infections caused by Zika virus, have been reported in several countries of South and South-eastern Europe in the past 20 years [27,28,29,30]. The detection of cases not associated with travel to endemic regions and the spread of these pathogens to geographical areas previously unfamiliar with them has been associated with factors directly connected to, or indirectly attributable to, climate change. Lower mean annual winter and higher spring and summer temperatures have all been associated with the increased risk for epidemic outbreaks. However, evidence is not always conclusive [31,32,33,34]. Vectors, in this case mosquitoes, replicate, and in general function above a threshold temperature of 10 °C [35]. With elevation of the temperature within tolerable limits, their replication cycle shortens and feeding has to increase, and this translates into increasing biting frequency. Taking into account that under moderately elevated temperatures virogenesis is also augmented, it can reasonably be assumed that global warming leads to increased pathogen transmission to the susceptible hosts [36,37]. However, historical examples of three prominent mosquito-borne diseases (malaria, yellow fever, and dengue) have challenged the significance of temperature compared to other environmental or human—inflicted modifications of the regional ecology [38].

Besides the greenhouse effect, the extreme weather phenomena recorded in association with global climate change influence ecosystems through increased precipitation, leading to shifts in relative humidity; also dependent on mean temperature and ground moisture. Once more, humidity appears to have conflicting effects on the spread of mosquito-borne diseases [39]. The increased relative humidity was associated with and increased survival rate of *Aedes albopictus*, but not *Culex* sp. in separate reports [31,40]. However, observed biting rates consistently relate to drier conditions [41,42]. Soil moisture is interrelated with regional vegetation cover and exerts a mixed effect on the transmission of WNV dependent on temperature variations [43]. Table 1 summarizes climate factors which have been implicated in the development of outbreaks of mosquito-borne emerging infectious diseases in Europe.

**Figure 1:**
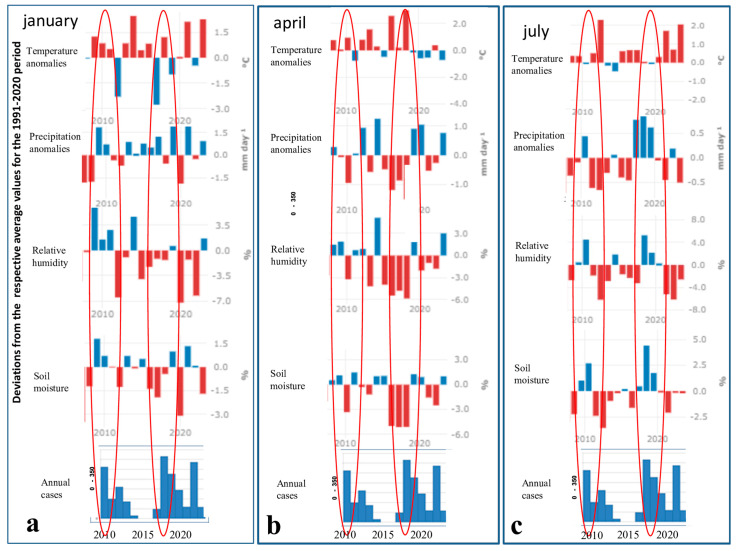
Mean monthly climatic anomalies and hydrological variables of Greece for the years 2018–2023 and their comparison with the intensity of annual local WNV epidemics (**a**) for January, (**b**) April, and (**c**) July. Data were extracted from the Copernicus Climate Change Service/ECMWF. Circles correlate the big epidemics of 2010 and 2018 with the climatic variables.

In 2010, a big WNV infection outbreak was recorded in Europe, particularly in the South-eastern part, both in humans as well as in equids. The summer of 2010 was characterised by exceptionally hot temperatures, with temperature elevations of more than 5 °C above the usual ones. It is presumed that the heat wave may indeed have contributed to the development of the epidemic [57]. Whether or not this association is always valid has not been addressed yet. Figure 1 correlates the average temperature, precipitation, relative humidity, and soil moisture anomalies of January, April, and July, representing winter, spring, and early summer, respectively, of each year in Greece from 2010 to 2023, compared with the average values during the reference period 1991–2020, to investigate potential associations with the intensity of each year’s epidemic. The two major epidemics of 2010 and 2018 in Greece since 2010 are highlighted by the oval shape and addressed separately.

## 5. Contribution of the Newly Translocated Arthropod Vectors to WNV Outbreaks in Europe

The migration, in fact, the transportation in tyres or with transport media, and the settling of the invasive tiger mosquitos (*Aedes* sp.) in the European continent sounded the alarm for public health authorities [58]. The two major factors ecologically favouring their local installation are considered to be climate warming and the increasing urbanization [59]. The establishment of these tropical mosquitos is considered, per se, to increase the risk for emerging infectious disease outbreaks due to their specific properties which clearly distinguish them from the currently circulating *Culex* sp. First of all, they are able to infestate urbanized areas, in which they and the eggs that they lay can survive considerably long. In terms of feeding habits, they preferentially bite humans and they will bite throughout the daytime, but do so mostly at dawn and dusk. Their most important characteristic is that they represent very well-adapted vectors to all the mosquito-borne viruses implicated in, and predicted to, further cause emerging infectious disease outbreaks (Chikungunya, West Nile, Dengue, Zika) [49]. Six invasive *Aedes* mosquito species have migrated to Europe since the 1970s: *Aedes aegypti*, *Aedes albopictus*, *Aedes japonicus*, *Aedes koreicus*, *Aedes atropalpus,* and *Aedes triseriatus*. However, the first pan-European surveillance effort organized in 2020 recorded the presence of two of them, *Aedes albopictus* and *Aedes japonicus* along with the native Culicidae [60]. *Aedes aegypti*, although present in the continent since the early 20th century, disappeared in the 60s, only to become reimported in 2008 in the area of Caucasus and the Black Sea [61]. For not fully apprehended reasons, however, despite its spreading west along the Black Sea coast, it is not yet established anywhere in the region in contrast to North America, probably due to other conditions, not presently fulfilled in Europe or even the properties and characteristics of the particular Mediterranean ecotype population of *Aedes aegypti*, clearly different from the American and Asian populations [62].

*Aedes albopictus*, on the other hand, originating from Southeast Asia, appeared in Albania in 1979, but finally settled in the Mediterranean Basin in 1990, when it was isolated again in a playground in Italy [50]. In contrast to *Aedes aegypti*, *Aedes albopictus* has demonstrated a remarkably widespread global distribution, now listed as one of the top 100 invasive species by the Invasive Species Specialist Group [51]. This differential adaptation is partly attributed to its increased tolerance of lower temperatures, that is, stronger adaptation to temperature instabilities [52], enabling it, theoretically regionally, to prevail and attenuate the installation of other invasive mosquitos. In comparison to its counterpart, *Aedes aegypti*, this species feeds on a wide range of hosts, thus theoretically presenting a serious public health threat as a bridge vector of zoonotic pathogens to humans [63].

Despite their potency to effectively transmit Flaviviridae and Togaviridae, in active mosquito surveillance systems which were implemented after outbreaks of West Nile fever mainly in South-eastern Europe, e.g., Greece, WNV was only detected in *Culex pipiens complex* mosquito pools in several instances [64,65]. Sixteen studies investigated the probability of different vectors’ WNV carriage in association with factors/locations affecting their breeding [66]. *Culex pipiens complex* was repeatedly identified as the key WNV vector, clearly indicating that the biological selection of the vector that will overtake the transmission of the pathogen is dependent on a wide, potentially circumstantial range of factors and has surely not been fully clarified yet.

## 6. The Impact of Socioeconomic Factors on Vector-Borne Diseases

Anthropogenic factors such as urbanization, human activities associated with the spreading of arthropod vectors outside their historical habitats, or other conditions with socioeconomic impact have, in several studies, been associated both with an amplified WNV presence in vectors and with increased incidence of WNV-associated disease [67]. Densely populated urbanized areas are associated with older infrastructure, impaired water drainage systems, and insufficient sanitation [68]. Lower-income communities are less aware of the public health risks that are inextricably linked to the creation of vector hotspots and do not demand, as firmly, pest control interventions [69]. Results from a study performed in Tennessee indicated, among others, that indeed vector abundance was higher in lower-income urban areas compared to higher-income ones [70].

During periods of austerity, governments may have to cut back on public health preservation efforts, such as spending on flood prevention measures, sanitation, and wastewater management, but also public education programs and health promotion [25]. The dysfunction of healthcare systems may additionally contribute to the aggravation of emerging infectious disease outbreaks. The beginning of the 21st-century era was characterised by a global financial recession. During Greece’s recent economic turmoil, from its initial manifestation in July 2010 until December 2011, that is, within a period of 18 months only, three infectious disease outbreaks took place. Aside from the increased mortality of the influenza epidemic that was recorded during the same period, the outbreaks included: an outbreak of West Nile virus infection in the summer and early autumn of 2010, with 197 reported patients with neuroinvasive disease and 35 total deaths, in the geographical area of Northern Greece; an outbreak of malaria in southern Greece during the summer of 2011 with 63 reported cases, of which 40 were considered autochthonous (non-imported); and an epidemic wave of new Human Immunodeficiency Virus cases with a 57.2% increase in newly diagnosed cases and a 1506.7% increase of newly diagnosed cases in injection drug users [71]. Environmental factors indisputably represent the leading cause of these epidemiological crises. However, the previously presented data can only support the theory that the austerity measures imposed upon the health sector, social services, and healthcare politics in Greece appear to have had a significant impact on public health.

## 7. West Nile Virus: An Endemic Pathogen in Greece

Before 2008, WNV outbreaks in Europe involved few cases and faded out very abruptly with minimal cases in the consecutive couple of years under very stringent surveillance [72].

Up until 2010, no human cases had been described in Greece. Seropositivity in studies was 1% among farmers [73]; the prevalence was significantly higher among animals, however, reaching 24% in birds [74]. Retrospective analysis of serum samples from inhabitants of Imathia, an area in Central Macedonia, which had a leading role in the majority of the outbreaks recorded in Greece from 2010 onwards, showed similarly a positivity rate of 1.02% [75].

**Figure 2:**
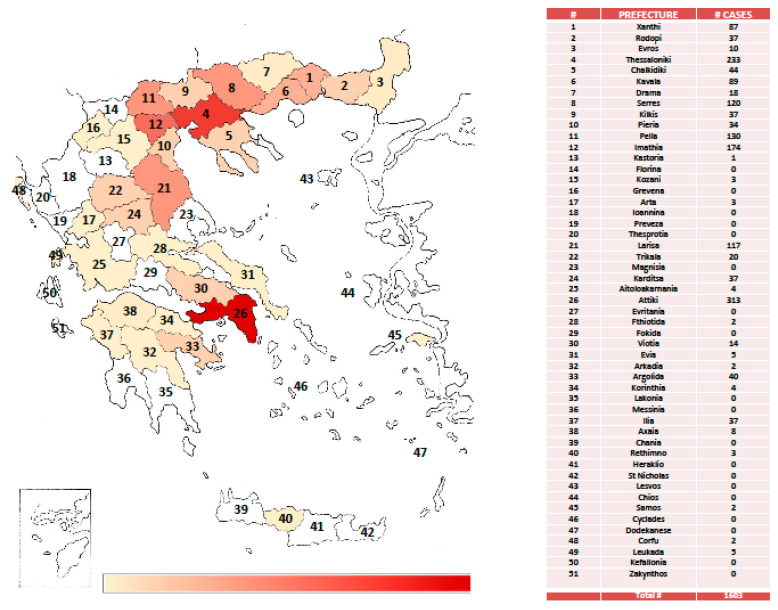
Map of Greece with total numbers of human WNV cases throughout the 13.5-year period 2010–2023, by prefecture. White colour represents prefectures where no human cases were observed.

In the summer of 2010, Greece experienced its first outbreak of West Nile virus infections, recording 262 cases in total and 35 deaths [76]. Since 2010, Greece has experienced annual outbreaks throughout, with variable intensity, with only a 2-year interval without any recorded cases (2015–2016) [77]. Seropositivity in the corresponding area of Greece (prefecture of Imathia and Pella) rose within weeks of the first outbreak from roughly 1.0 to 5.8% [78]. In these 14 years, from 2010 until now, a total of 1603 verified cases of West Nile fever have been reported in Greece in a total of 32/51 prefectures, progressively expanding (Figure 2). It needs to be stressed that the number of verified recorded cases each year is only a small proportion of the actual ones and refers to the patients who develop persistent symptomatology or present with a more severe clinical condition qualifying for further investigation. Moreover, the mapping of the geographical distribution of the cases may not completely coincide with the actual site of infection, due to travelling and people translocation. Specific areas, mainly in the northern and central mainland, are considered WNV “hot spots” as they consistently record confirmed WNF cases during the annual epidemics [79].

Integrated surveillance protocols, implemented in Greece since the first outbreak, included obligatory notification of verified human cases and active sentinel surveillance of vectors from mosquito traps and equids, indicating the steady presence of the virus throughout, irrespective of whether human cases were recorded during a particular year (2015–2016 were theoretically free of recorded, at least, WNV cases) [80].

The majority of the outbreaks recorded in Europe in the past two decades (since 2008), including the annual outbreaks in Greece, were attributed to the neurovirulent strain belonging to lineage 2, which was imported to Europe from Africa and has since then become established in the area [81]. A recently published epidemiological report from Greece’s National Public Health Organization (EODY) and the National Reference Laboratories, collecting and molecularly characterising viral strains detected in patients, blood donors mosquitoes, horses, and birds since 2010, reported that all but one WNV sequence clustered into the Central European subclade of WNV lineage 2 [79]. A viral strain belonging to the Eastern European/Russian subclade of WNV lineage 2 was detected in a patient from north-eastern Greece in 2018, indicating the introduction of a new virus in the area [82]. Clearly, the formerly considered to be an emerging infectious disease pathogen has become an endemic pathogen, well established in the region.

## 8. Comparison of Climatic Anomalies, Hydrological Variables, and Annual WNF Outbreaks in Greece

Annual data extracted from the Copernicus Climate Change Service/ECMWF concerning climatic and hydrological parameters were juxtaposed to the annual WNF epidemics from the Greek National Centre for Disease Control Registry, [National Public Health Organization-EODY, annual WNV surveillance reports (2010–2023)]. January, April, and July were chosen to represent winter, spring and summer, respectively. Temperature and precipitation anomalies, as well as relative humidity and soil moisture deviations, were compared to the intensity of the annual WNF epidemic waves (Figure 2). Interestingly, the two more pronounced WNF outbreaks of 2010 and 2018, of 262 and 316 WNF cases, respectively, were associated with a warmer winter; a warmer, drier spring; and a more humid, not necessarily warmer, summer (oval shapes). These results suggest that indeed global warming may facilitate vector hibernation, and warm, drier springs may accelerate earlier WNV transmission, resulting in a higher circulation during the following months, while increased humidity during the summer contributes to the peaking of mosquito-borne diseases, probably by accelerating the vectors’ reproduction [83,84].

## 9. Conclusions

WNV has been established in the area of South, South-eastern and, lately, Western and Central Europe, and is spreading towards its northern part with the aid of climate change, which in turn facilitates its potential to survive and spread in previously inhospitable regions [85]. Outbreaks of locally acquired infections are recorded annually, rendering WNV the leading cause of arboviral disease, not only in the US, but also in Europe [86,87]. According to the report of the European Food Safety Authority and the European Centre for Disease Prevention and Control concerning the results of zoonoses’ monitoring and surveillance activities, WNV together with Listeriosis is the most severe zoonotic disease, with the most hospitalisations and the highest morbidity [88].

Greece ranks among the countries with the highest annual notification rates with significant fluctuations, or rather, with an irregular wavy pattern in the intensity of its outbreaks. Annual reported symptomatic cases in the years from 2010 to 2022 varied from 0 to 311 with a notification rate for West Nile cases with neuroinvasive disease ranging from 0 to 2.2 per 100,000 population. The associated mortality rate ranged from 9% to 40%. Initiation of each year’s epidemic fluctuated and was recorded from as early as May 31st with the detection of the first annual case up to as late as August 5th. Years with a higher epidemiological burden were usually characterised by an early start of the epidemic [79].

Predicting the severity of each year’s epidemic seems unrealistic as a variety of factors exert their effect and interrelate in a highly complex manner. An intense previous season justifies public health authorities’ alertness. Recording of WNF cases in a geographical area during the summer to early autumn of a specific year carries a risk of recurrence of WNV infections in the same region of up to 50% for the next month of the season and 20% for the subsequent year [89].

Climate factors favouring potential outbreaks are more versatile and affect geographical regions relative to their native climatic conditions. In an epidemiological study from Spain, the combination of high precipitation rates, soil moisture, and a warmer spring provided more favourable environmental conditions for mosquitoes to breed and transmit WNV [90], in agreement with our analysis.

As the virus has gained endemicity in the region and has already been steadily circulating for more than a decade, perhaps the most reliable tool to predict the next epidemic at this point is to monitor mosquito-borne viral activity prior to the initiation and throughout each year’s candidate season. Greece has one of the most organized WNV surveillance programs [91]. Increases in the mosquito viral positivity rates aid public health authorities to promptly adopt emergency measures to halt the potential development of an outbreak, such as the implementation of vector control measures and the use of biocides.

WNV and other flaviviruses have expanded their distribution significantly and are expected to increasingly burden public health systems in the future. It therefore is of particular significance to develop reliable outbreak prediction tools adjusted to each particular pathogen’s ecology that will be implemented in order to limit the spread of the virus and lessen the impact of the forthcoming epidemic.

## Figures and Tables

**Table 1:** Factors affecting the intensity of WNV outbreaks in Europe.

Environmental Factors				References
Mean Temperature	Winter	Spring	Summer	
Type of association	negative	positive	positive	[31,32,33,34,37,44]
Proposed mechanism	affects vector survival, depending on the vector	increases biting rate, increases host-to-vector transmission probability, accelerates mosquito viral incubation period, potentially enhances oviposition activity	affects distribution, behaviour and survival of the mosquito vector, reduces incubation period in the competent vectors and affects host distribution and behaviour very high temperatures reduce mosquito survival	
Relative Humidity/Precipitation	Winter	Spring	Summer	
Type of association	negative	positive/negative	negative	[34,36,45,46,47]
Proposed mechanism	affects vector survival	increases the standing water resources at the beginning of the hot season/consequence of favourable higher temperatures- drought creates stagnant water	drought leads to increases in mosquito numbers and increases biting rate	
Ground Moisture/Vegetation	Winter	Spring	Summer	
Type of association	positive (when followed by a decrease in spring/summer)	depending on mean temperatures	positive	[43,46]
Proposed mechanism	variations influence the presence and size of the WNV vector populations	inconclusive	favours vector abundance	[48]
Vector-Associated Factors	Current Situation in Europe		Effect on Potential Outbreak	
Installation/availability of potent arthropod vectors	installation and endemicity in the Mediterranean Basin/Europe of *Aedes* mosquitoes, mainly *albopictus*		positive (pros: very potent vectors, are able to infestate urbanized areas, bite throughout the daytime, contras: preferentially bite humans)	[49,50,51,52]
Hosts/reservoirs	climate change favours vector abundance, geographic location on the route of migratory birds from Africa		positive	[53]
Vector and viral overwintering	evidence of enzootic circulation, viral overwintering in non-migratory birds, evidence of viral overwintering in mosquitoes		positive	[23,24,54,55]
Socioeconomic Factors	Current Situation in Europe		Effect on Potential Outbreak	
Economic crisis	coincided with the outbreak in 2010		negative correlation of the Gross Domestic Product with WNV infections	[56]
